# Death of Dr. Pritchard and Dr. Townes

**Published:** 1849-05

**Authors:** 


### DEATH OF DR. PRITCHARD AND DR. TOWNES.

The death of these eminent men is announced in the late English journals. Dr. Pritchard, by his great work on the natural history of Man, and Dr. Townes, by his writings on Chemistry, have made their names familiar to American readers. Dr. Townes was still comparatively young, and promised to confer great benefits upon the science to which he was ardently devoted.

