# Correction

**Published:** 1849-06

**Authors:** 


### CORRECTION.

In the obituary notice of Dr. Prichard and Dr. Fownes, in our last number, an error in both names was overlooked by the proof-reader. They are here correctly given.

